# Linear Factor Analytic Thurstonian Forced-Choice Models: Current Status and Issues

**DOI:** 10.1177/00131644231205011

**Published:** 2023-10-30

**Authors:** Markus T. Jansen, Ralf Schulze

**Affiliations:** 1 University of Wuppertal, Germany

**Keywords:** Thurstonian modeling, forced-choice format, item response theory, structural equation modeling

## Abstract

Thurstonian forced-choice modeling is considered to be a powerful new tool to estimate item and person parameters while simultaneously testing the model fit. This assessment approach is associated with the aim of reducing faking and other response tendencies that plague traditional self-report trait assessments. As a result of major recent methodological developments, the estimation of normative trait scores has become possible in addition to the computation of only ipsative scores. This opened up the important possibility of comparisons between individuals with forced-choice assessment procedures. With item response theory (IRT) methods, a multidimensional forced-choice (MFC) format has also been proposed to estimate individual scores. Customarily, items to assess different traits are presented in blocks, often triplets, in applications of the MFC, which is an efficient form of item presentation but also a simplification of the original models. The present study provides a comprehensive review of the present status of Thurstonian forced-choice models and their variants. Critical features of the current models, especially the block models, are identified and discussed. It is concluded that MFC modeling with item blocks is highly problematic and yields biased results. In particular, the often-recommended presentation of blocks with items that are keyed in different directions of a trait proves to be counterproductive considering the goal to reduce response tendencies. The consequences and implications of the highlighted issues are further discussed.

Many basic and applied empirical research efforts in psychology, as well as other social and behavioral sciences, include measurements of latent traits with self-reports. Pertinent examples can be found in psychiatric (Carey et al., 2004; Cochrane-Brink et al., 2000), educational, and legal applications (e.g., Holden & Passey, 2010), or in the context of research on predicting vocational performance (Barrick & Mount, 1991; Tett et al., 1991). Given that many psychological constructs and behavioral tendencies (e.g., being emotionally stable) are expected to be efficiently accessible via introspection at the individual level, self-assessments appear to be a perfectly viable way of assessing the targeted constructs. An individual’s self-reported strength of agreement on a rating scale (e.g., from “totally disagree” to “totally agree”) to a statement (e.g., “In difficult situations I stay calm”) may serve as an indicator to estimate scores of emotional stability, for example. Of course, many such Likert-type items would generally be needed as indicators to arrive at a sufficiently precise estimate of the individuals’ scores on the targeted construct and to be able to make interindividual comparisons.

A quite common problem with many forms of self-assessments, including the above-mentioned Likert-type items in particular, is their susceptibility to a myriad of response biases. Their proneness to such response distortions depends on the context as an abundance of empirical research shows, and it is particularly prevalent in, but not limited to, high-stakes contexts (e.g., Ziegler et al., 2012). Typical response biases include the tendency to agree to a statement irrespective of its content (acquiescence bias), the tendency to very strongly agree or disagree (extreme responding), to give responses that are expected to be socially approved (social desirability bias, e.g., Cronbach, 1946; Jackson & Messick, 1958; Paulhus, 2002), and faking (Ziegler et al., 2012). In addition to response biases, other methodological issues also prevail with Likert-type items. They include the question if interpretation and use of a given response scale are consistent both within and between individuals. Different approaches to mitigate the influences of response biases and faking have been proposed, but most of them are not without problems as they are associated with new challenges like more complicated item-phrasing or the reduction of reliability and construct validity (Paulhus & Vazire, 2007), for example.

The present article focuses on one particular methodological approach to deal with the majority of these problems and challenges, namely the forced-choice design (FC). The main characteristic of the FC approach is that the respondents are required (“forced”) to make a choice between a set of stimuli (e.g., statements) with respect to a given criterion (e.g., the degree of fit of a statement as a descriptor of oneself). In contrast to Likert-type items, individual statements are not rated on a scale. Instead, comparisons between stimuli are required. Usually, two or more stimuli are presented in blocks (see Figure 1). A block may consist of only two stimuli, as is the case in Panel A of Figure 1. In this paired comparison, the respondents compare the two statements as descriptors of themselves and choose the better one, even if none or both of the statements provide a very good description of the person. It is also possible and, in fact, more common to present more than two stimuli for a simultaneous comparison as is the case in Panel B of Figure 1. Here, respondents are asked to rank the statements by choosing one statement that fits the best and one statement that fits the least as a description of oneself. By implication, these two decisions result in a full ranking of all three stimuli. Note that individuals are not asked to respond to each of the items separately but to give a preference ranking over the combined block of statements.

**Figure 1.**
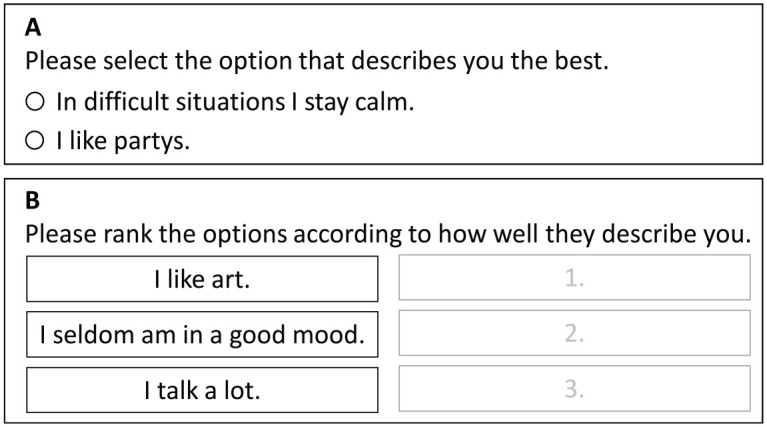
Examples for the Forced-Choice Format. Panel A is an example for a paired comparison, where a person must choose one of the items. Panel B is an example for the ranking of a triplet.

If interindividual differences are of interest, one major drawback of FC designs is the limited information that the resulting data provides. Traditional scoring methods for such data only lead to so-called ipsative scale scores, which cannot reasonably be compared across respondents. This results from the fact that if the stimuli are assigned their ranks as scores, the same total number of points is distributed between stimuli within an FC block and for each block of the same size. Therefore, if such scores are added up within one block and across all blocks, the total score of a test is the same for every person. As a result, the usual score interpretations that refer to between-subject comparisons and classic psychometric analysis are impossible (for further discussion, see Baron, 1996).

To overcome the limitations in interpretation with ipsative scoring for interindividual differences, two general model frameworks are considered. They potentially allow for the estimation of so-called normative scores, that is, scores that allow for interindividual score interpretations. The first model framework concentrates on the ideal point model (Coombs, 1960; see also Stark et al., 2005), whereas the second is based on linear factor analysis models or the dominance-response process (see Brown, 2016). We will exclusively focus on the dominance-response process as it is more commonly used. In dominance-response processes, it is assumed that each item is linearly related to one factor. The higher a respondent’s score on the factor, the stronger the agreement to the item should be. The common model framework for the dominance-response process is Thurstone’s Law of Comparative Judgment (LoCJ; Thurstone, 1927, 1931). The original and typical use of the LoCJ is to scale items and other stimuli according to specific criteria. Unfortunately, initial estimation methods for Thurstone’s models were unfeasible for practical applications (Maydeu-Olivares, 1999; Yao & Böckenholt, 1999). The development of better technical resources and limited information estimation methods, though, made a confirmatory factor analytic (CFA) method for item scales (Maydeu-Olivares & Böckenholt, 2005) and item response theory (IRT) models for person scores possible (Brown & Maydeu-Olivares, 2011; Maydeu-Olivares & Brown, 2010). As a result, both stimuli scale values and respondent scores can be estimated in this framework, which makes it particularly attractive for research and applications.

Alas, several studies (e.g., Bürkner, 2022; Bürkner et al., 2019; Frick et al., 2023; Schulte et al., 2021) have already shown that Thurstonian modeling is not without issues and challenges; the review, identification, and extended specification of which will be the focus of the current study. This will be done in three sections. In the first section, current Thurstonian models that all employ binary outcome variables will be specified and examined. In the second section, the status of current Thurstonian modeling and associated prevailing issues as well as open questions are reviewed and summarized. Finally, the results and consequences of the findings of the previous section are discussed. Hence, the aims of the current paper are to (a) comprehensively review the present status of Thurstonian forced-choice models as well as some of their variants, (b) provide an overview of the list of known current limitations and shortcomings and add to this list new entries, and (c) point to routes of improvement to address these issues. With the current article, we aim to lay the foundation for a discussion on how to proceed with the study of Thurstonian models.

## Thurstonian Models

The analysis of paired comparison and ranking data uses latent paired comparisons. As the coding of responses has been explained repeatedly in the pertinent literature, we refer to the important works of Maydeu-Olivares and Böckenholt (2005) as well as Brown and Maydeu-Olivares (2011).

In Thurstonian models, it is assumed that each comparison provokes a discriminative process where two scale values, also called latent utilities, are compared to each other. For the latent response process, consider a scale value *t_i_* (for stimulus *i*) on a latent utility continuum of a respondent. The difference between the latent utilities determines if stimulus *i* or *j* is chosen (i.e., preferred, if preference is the criterion). The entire process is not observable, but the response $y_{l}$ that results from this process is, and it is coded as

(1) $$y_{l}=\left\{ \begin{array}{c} 1\;\mathrm{if}\;t_{i}\geq t_{j}\\ 0\;\mathrm{if}\;t_{i}<\;t_{j} \end{array}.\right.$$

Also, the difference between the processes $y_{l}^{*}=t_{i}-t_{j}$ is not observable, but the relationship between unobservable $y_{l}^{*}$ and observed $y_{l}$ is

(2) $$y_{l}=\left\{ \begin{array}{c} 1\;\mathrm{if}\;y_{l}^{*}+\;e_{l}\;\geq 0\\ 0\;\mathrm{if}\;y_{l}^{*}\;+\;e_{l}\;<0 \end{array}\right..$$

Note that there is now an error term $e_{l}$ for each comparison to cover the issue of transitivity that may arise in the present context. Transitivity is given for three stimuli A, B, and C when A is preferred over B, B over C, and also A over C. The latter choice is implied in rankings but may not be observed in paired comparisons. Respondents are absolutely free in a paired comparison design to choose C over A in the example, thereby violating transitivity. This is why the term $e_{l}$ is included in Equation 2. A major consequence of a (full) ranking design is that all responses are always transitive. Thus, the error term $e_{l}=0$ for every comparison in a ranking design but not in paired comparison designs.

There are several Thurstonian linear factor analytic models that differ in focus on estimation (item-centered vs. person-centered) and designs (full vs. block). In the following section, the different types of Thurstonian models will be specified and examined, and their usability as well as differences will be pointed out.

### Simple Thurstonian Model

The simple Thurstonian model was introduced by Maydeu-Olivares and Böckenholt (2005). As it is based on Thurstone’s LoCJ, its focus of estimation is on the item scale values. Writing all latent differences $y_{l}^{*}$ between the latent utilities in vector-matrix form yields

(3) $$y^{*}=\mathrm{At}+e$$

for $y^{*}$ , which is a $\tilde{n}\times 1$ vector. The n×1 vector of the latent utilities is t , and the $\tilde{n}\times n$ design matrix is A , where the rows of A correspond to the paired comparisons and the columns correspond to the choice alternatives. Finally, e is a $\tilde{n}\times 1$ vector of uncorrelated random errors terms. As a consequence, the covariance matrix $\Omega^{2}$ of the residuals is diagonal. For example, given *n* = 4, the design matrix is

(4) $$A=\left[\begin{array}{cccc} 1 & -1 & 0 & 0\\ 1 & 0 & -1 & 0\\ 1 & 0 & 0 & -1\\ 0 & 1 & -1 & 0\\ 0 & 1 & 0 & -1\\ 0 & 0 & 1 & -1 \end{array}\right].$$

**Figure 2.**
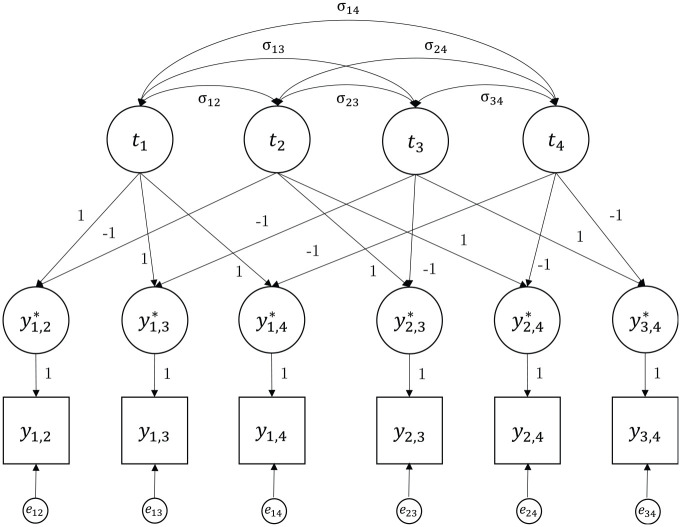
Example of a Covariance Structure of a Simple Thurstonian Model for n = 4 Items.

Generally, it is assumed that in the population of respondents, the latent utilities follow a multivariate normal distribution (Maydeu-Olivares & Böckenholt, 2005; Thurstone, 1927), that is

(5) $$t\sim N(\mu_{t},\sum_{t}),$$

where $\mu_{t}$ is the mean vector, and $\sum_{t}$ is the variance-covariance matrix of latent utilities. Equation 6 includes the latent utilities in a structural equation model with binary indicators as follows

(6) $$\mu_{y^{*}}=A\mu_{t},\mathrm{and}\;\sum_{y^{*}}=A\sum_{t}A\prime +\Omega^{2},$$

where $\mu_{y^{*}}$ is a vector with the mean latent differences, and $\sum_{y^{*}}$ is the variance-covariance matrix of the $\tilde{n}$ latent differences. To estimate such models, the thresholds and tetrachoric correlations of the dichotomized normal variables must be computed, and certain constraints on the covariance matrix $\Omega^{2}$ have to be considered (B. Muthén, 1978). Alternatively, the latent differences are standardized to $z^{*}=D(y^{*}-\mu_{y^{*}})$ , with $D={\left[\mathrm{diag}(\sum_{y^{*}})\right]}^{-1/2}$ following a multivariate normal distribution, now with $\mu_{z^{*}}=0$ . To identify the model, the thresholds and the matrix of tetrachoric correlations $P_{z^{*}}$ must then be constrained instead of the covariance matrix $\Omega^{2}$ . The relationship between observed responses and standardized latent differences is one of the $\tilde{n}$ thresholds $\tau_{l}$

(7) $$y_{l}=\{\begin{array}{ccc} 1 & \mathrm{if} & z_{l}^{*}\geq \tau_{l}\\ 0 & \mathrm{if} & z_{l}^{*}<\tau_{l} \end{array},$$

where the vector of thresholds is $\tau =-\mathrm{DA}\mu_{t}$ , and the tetrachoric correlation matrix is

(8) $$P_{z^{*}}=D(\sum_{y^{*}})D=D(A\sum_{t}A\prime +\Omega^{2})D$$

(Maydeu-Olivares & Böckenholt, 2005; B. Muthén, 1978). Figure 2 shows an example of a simple Thurstonian model for *n* = 4.

The simple Thurstonian model allows for the estimation of the mean latent utilities (item scale values) for item scaling and the test of Thurstone’s cases (especially Cases II, III, and V; Thurstone, 1927, 1928). For example, for Thurstone’s Case V model, it is assumed that all latent utilities t are uncorrelated and that the variances of the latent utilities are equal. If such constraints were used for $\sum_{t}$ , then (comparative) tests of the restrictions implied by different Thurstone cases could be conducted, and an assessment of the relative fit would be possible (for more details, see Maydeu-Olivares & Böckenholt, 2005).

### Thurstonian Factor Model

**Figure 3.**
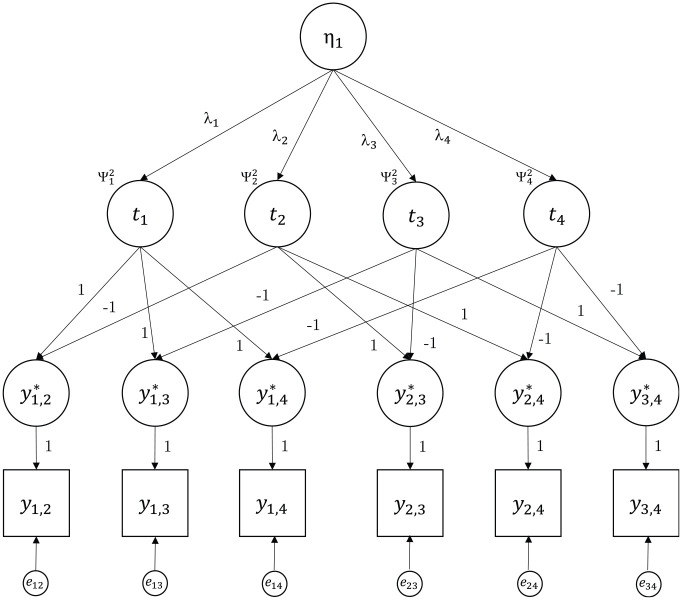
Example of a Covariance Structure of a Thurstonian Factor Model for n = 4 and m = 1.

Instead of a correlated model as shown in Figure 2, there may be a known structure on the *n* items implied by a higher-order model with dichotomous indicators as shown in Figure 3, for example. Such a model is called the Thurstonian factor model (Maydeu-Olivares & Böckenholt, 2005). Let *m* be the number of such common factors (also called latent traits). The *n* latent utilities in t can then be expressed as

(9) $$t=\mu_{t}+\Lambda \eta +\varepsilon ,$$

where Λ is the n×m matrix of factor loadings of the latent utilities on the latent traits, η is a m×1 vector of the latent traits, and ε is a n×1 vector of unique factors (error term). All common factors have a mean of zero and variances of unity but may be correlated as allowed for in an m×m correlation matrix Φ . The unique factors also have mean zero but are uncorrelated. Thus, the variance-covariance matrix $\Psi^{2}$ of error terms is diagonal. With Equations 3 and 9, the latent differences are

(10) $$y^{*}=A(\mu_{t}+\Lambda \eta +\varepsilon )+e=A\mu_{t}+A\Lambda \eta +A\varepsilon +e,$$

which results in the mean and covariance structure

(11) $$\mu_{y^{*}}=A\mu_{t},\mathrm{and}\;\sum_{y^{*}}=A(\Lambda \Phi \Lambda \prime +\Psi^{2})A\prime +\Omega^{2}.$$

The implied tetrachoric correlations follow from Equations 8 and 11 to be

(12) $$P_{z^{*}}=D(\sum_{y^{*}})D=D(A(\Lambda \Phi \Lambda \prime +\Psi^{2})A\prime +\Omega^{2})D$$

with the same relationship between standardized latent differences and observed responses as in Equation 7 and vector of thresholds $\tau =-\mathrm{DA}\mu_{t}$ . Figure 3 gives an example for a Thurstonian factor model with *n* = 4 and a single factor *m* = 1.

For ranking data, intransitive responses are not possible. As a consequence, all model equations also hold in a ranking design, but with e=0 and $\Omega^{2}=0$ . In addition, rankings include only a subset of all possible paired comparisons, so the number of degrees of freedom must be adjusted, as there are

(13) $$r=\frac{n(n-1)(n-2)}{6}$$

redundancies for the tetrachoric correlations and thresholds (Maydeu-Olivares, 1999; Maydeu-Olivares & Böckenholt, 2005). Therefore, *r* must be subtracted from the number of degrees of freedom reported by a structural equation modeling software program, and fit indices must be adjusted accordingly.

### Use and Identification of Simple Thurstonian and Thurstonian Factor Models

One latent utility mean must be fixed to identify the model, for example, $\mu_{n}=0$ . All other latent utility means are estimated relative to the fixed value. Note that the identification constraints are not unique so that the model parameters are always of relative nature. For all simple Thurstone models, at least one variance must additionally be fixed to $\sigma_{n}^{2}=1$ , for example. Specifically, the constraints proposed by the different Thurstone cases on the variance-covariance matrix of latent utilities $\sum_{t}$ must be set (see Maydeu-Olivares & Böckenholt, 2005). To estimate the latent item means, all thresholds must also be fixed (e.g., to zero). Furthermore, one factor loading must be fixed in Thurstonian factor models with only one latent trait (e.g., $\lambda_{1}=1$ ). This loading constraint would not be necessary if more than one latent trait is included in the model (Maydeu-Olivares & Brown, 2010). Finally, one variance of one latent utility and the variances of the latent traits need to be fixed to unity. These last constraints set the scales of the factor loadings and the unique variances.

Simple Thurstonian models and Thurstonian factor models are used whenever the means of the latent utilities or latent traits are of interest. Prototypical cases would be given by applications of item scaling, where item properties on a latent trait (Maydeu-Olivares & Böckenholt, 2005) or their social desirability (Jansen & Schulze, 2023a), for example, are of interest. In addition to item scaling, trait scaling may also be of interest. Disregarding test construction studies, item and trait scaling are rather rare in psychological research. In most research efforts, the item properties are not of primary interest, but interest lies much more heavily on individual scores and individual differences. Nevertheless, item scaling methods are not exclusively but particularly important for applications of Thurstonian unrestricted thresholds and IRT models, which are reviewed and discussed in what follows.

### Thurstonian Factor Model With Unrestricted Thresholds

If latent utilities are not of interest, they are fixed to zero, and the intercepts in the model are freely estimated instead. According to Equations 3 and 9, the latent differences are

(14) $$y^{*}=\mathrm{At}+e,\;t=\mu_{t}+\Lambda \eta +\varepsilon .$$

The corresponding $\tilde{n}$ intercepts are defined as −γ . They are constrained for identification of the latent utility means as

(15) $$\gamma =-A\mu_{t}.$$

Accordingly, the model with unconstrained intercepts is defined by

(16) $$y^{*}=-\gamma +\mathrm{At}+e,\;t=\Lambda \eta +\varepsilon .$$

The threshold structure is τ=Dγ and unconstrained as it is a rescaling of γ by D . Thurstonian factor models with unrestricted thresholds are hardly ever of particular interest. However, this model type is important for the use of Thurstonian IRT models. The Thurstonian factor models with unrestricted thresholds are considerably less constrained than the Thurstonian factor models. More importantly, with unconstrained thresholds (and restricted means), the consideration of individual differences would be possible. In a paired comparison design, factor scores can indeed be estimated. However, if only stimuli rankings are used, factor scores estimation is not possible anymore in the present framework. This is due to the non-positive residual variances of the categorical indicators with e=0 and $\Omega^{2}=0$ .

### Thurstonian IRT Model

The main advantage of the unconstrained factor model is the possibility of a straightforward reparameterization into a first-order model. The reparameterized model can equivalently be expressed as an IRT model (Brown & Maydeu-Olivares, 2011). For instance, Equation 16 can be reparameterized to

(17) $$y^{*}=-\gamma +A(\Lambda \eta +\varepsilon )+e=-\gamma +A\Lambda \eta +A\varepsilon +e=-\gamma +\overset{⌣}{\Lambda }\eta +\overset{⌣}{\varepsilon }$$

with $\overset{⌣}{\varepsilon }=A\varepsilon +e$ and $\mathrm{cov}(\;\overset{⌣}{\varepsilon })=\overset{⌣}{\Psi^{2}}=A\Psi^{2}A\prime +\Omega^{2}$ , where $\overset{⌣}{\Lambda }=A\Lambda$ is a $\tilde{n}\times m$ matrix. Assuming a single latent trait, so that *m* = 1 and *n* = 3, it would be

(18) $$\overset{⌣}{\Lambda }=\left(\begin{array}{ccc} 1 & -1 & 0\\ 1 & 0 & -1\\ 0 & 1 & -1 \end{array}\right)\left(\begin{array}{c} \lambda_{1}\\ \lambda_{2}\\ \lambda_{3} \end{array}\right)=\left(\begin{array}{c} \lambda_{1}-\lambda_{2}\\ \lambda_{1}-\lambda_{3}\\ \lambda_{2}-\lambda_{3} \end{array}\right),$$

and with *m* = 3 and *n* = 3, for example, it is

(19) $$\overset{⌣}{\Lambda }=\left(\begin{array}{ccc} 1 & -1 & 0\\ 1 & 0 & -1\\ 0 & 1 & -1 \end{array}\right)\left(\begin{array}{ccc} \lambda_{1} & 0 & 0\\ 0 & \lambda_{2} & 0\\ 0 & 0 & \lambda_{3} \end{array}\right)=\left(\begin{array}{ccc} \lambda_{1} & -\lambda_{2} & 0\\ \lambda_{1} & 0 & -\lambda_{3}\\ 0 & \lambda_{2} & -\lambda_{3} \end{array}\right).$$

In both cases,

(20) $$\begin{array}{c} \overset{⌣}{\Psi^{2}} \end{array}=\left(\begin{array}{ccc} \psi_{1}^{2}+\psi_{2}^{2}+\omega_{1}^{2} & & \\ \psi_{1}^{2} & \psi_{1}^{2}+\psi_{3}^{2}+\omega_{2}^{2} & \\ -\psi_{2}^{2} & \psi_{3}^{2} & \psi_{2}^{2}+\psi_{3}^{2}+\omega_{3}^{2} \end{array}\right).$$

Both models in Equations 16 and 17 are equivalent, as Equation 17 is simply a reparameterization. Therefore, both models have the same (but reparameterized) tetrachoric correlation matrix:

(21) $$P_{z^{*}}=D(A\Lambda \Phi \Lambda \prime A\prime +A\Psi^{2}A\prime +\Omega^{2})D=D(\overset{⌣}{\Lambda }\Phi \overset{⌣}{\Lambda }\prime +{\overset{⌣}{\Psi }}^{2})D.$$

Let Φ(x) be a standard normal distribution function at *x*, $\gamma_{l}$ the threshold for $y_{l}$ , ${\overset{⌣}{\lambda }}_{1}^{\prime }$ the vector of factor loadings, and ${{\overset{⌣}{\psi }}_{l}}^{\;2}$ the variance of the binary response. Then the item characteristic function (ICF) for the binary response variable for items *i* and *j* is given by

(22) $$\mathrm{Pr}(y_{l}=1|\eta )=\Phi \left(\frac{-\gamma_{l}+\overset{⌣}{\lambda_{l}\prime }}{\sqrt{\overset{⌣}{{\psi_{l}}^{2}}}}\right).$$

It is the ICF of a normal ogive model except that ${\overset{⌣}{\Lambda }}_{1}^{\prime }$ and ${{\overset{⌣}{\psi }}_{l}}^{\;2}$ are structured and that the ICFs are not independent (Brown & Maydeu-Olivares, 2011; Maydeu-Olivares & Brown, 2010). With only one latent trait, that is *m* = 1, Equation 22 specifically is

(23) $$\mathrm{Pr}(y_{l}=1|\eta )=\Phi \left(\frac{-\gamma_{l}+\overset{⌣}{\lambda_{l}}\eta }{\sqrt{\overset{⌣}{{\psi_{l}}^{2}}}}\right)=\Phi \left(\frac{-\gamma_{l}+(\lambda_{i}-\lambda_{j})\eta }{\sqrt{\psi_{i}^{2}+\psi_{j}^{2}+\omega_{l}^{2}}}\right).$$

However, if *m* > 1, then for each comparison, it is

(24) $$\mathrm{Pr}(y_{l}=1|\eta_{a},\eta_{b})=\Phi \left(\frac{-\gamma_{l}+\lambda_{i}\eta_{a}-\lambda_{j}\eta_{b}}{\sqrt{\psi_{i}^{2}+\psi_{j}^{2}+\omega_{l}^{2}}}\right).$$

Expressed in intercept and slope notation it follows from,

(25) $$\alpha_{l}=\frac{-\gamma_{l}}{\sqrt{\psi_{i}^{2}+\psi_{j}^{2}\omega_{l}^{2}}},\beta_{i}\frac{\lambda_{i}}{\sqrt{\psi_{i}^{2}+\psi_{j}^{2}+\omega_{l}^{2}}},\beta_{j}\frac{\lambda_{j}}{\sqrt{\psi_{i}^{2}+\psi_{j}^{2}+\omega_{l}^{2}}},$$

that, when *m* = 1, it is

(26) $$\mathrm{Pr}(y_{l}=1|\eta )=\Phi \left(\alpha_{l}+(\beta_{i}-\beta_{k})\eta \right),$$

whereas with *m* > 1, for each comparison, it is

(27) $$\mathrm{Pr}(y_{l}=1|\eta_{a},\eta_{b})=\Phi \left(\alpha_{l}+\beta_{i}\eta_{a}-\beta_{k}\eta_{b}\right).$$

**Figure 4.**
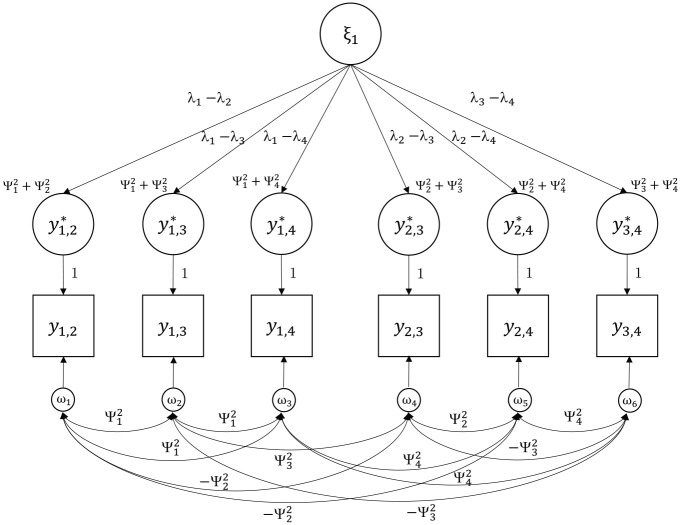
Example of a Covariance Structure of a Thurstonian Item Response Theory Model for n = 4 and m = 1.

Figure 4 shows an example for a Thurstonian IRT model with *n* = 4 and a single factor *m* = 1.

To identify the model, the variances of the latent traits and one error variance (uniqueness) need to be fixed to unity in the reparameterized model. The main advantage of the Thurstonian IRT model is the fact that the error variances are now non-zero and positive even for ranking designs. This allows for the estimation of the latent trait scores. From a practical viewpoint, it is also noted that IRT model estimation is faster than the estimation of Thurstonian factor models via CFA methods.

### Latent Trait Estimation

Following the estimation of the IRT model parameters, latent trait scores for each individual can also be estimated using their pattern of binary outcome responses. Generally, the maximum a posteriori (MAP) estimation is used (Brown & Maydeu-Olivares, 2011), which maximizes the mode of the posterior distribution of the latent traits. When IRT scores are obtained with the MAP method, the posterior test information for each respondent at each point MAP estimate is evaluated. The empirical reliability for the MAP scores can be calculated by (Brown & Maydeu-Olivares, 2011)

(28) $$\rho =\frac{\sigma^{2}-{\bar{\sigma }}_{\mathrm{error}}^{2}}{\sigma^{2}}$$

or by (Brown & Maydeu-Olivares, 2018)

(29) $$\rho =\frac{\sigma^{2}}{\sigma^{2}+{\bar{\sigma }}_{\mathrm{error}}^{2}},$$

where $\sigma^{2}$ is estimated using the variance of the scores, and ${\bar{\sigma }}_{\mathrm{error}}^{2}$ is estimated by

(30) $${\bar{\sigma }}_{\mathrm{error}}^{2}(\hat{\eta })=\frac{1}{N}\sum_{j=1}^{N}S{E^{2}}_{\mathrm{estimate}},$$

where $S{E^{2}}_{\mathrm{estimate}}$ are the squared standard errors of each estimate. This procedure assumes local independence between paired comparisons. However, the uniquenesses are structured and not independent. Alternatively, scores can be estimated with a genuine likelihood approach (Yousfi, 2019).

### Summary

So far, three types of Thurstonian models have been specified: The first one is the simple Thurstonian model with correlated latent variables for the items. This model can be used to estimate item properties and test Thurstone’s cases. The second one includes Thurstonian factor models that are of higher order with latent traits as the second-order factors. These models can be used to estimate item or trait properties. Only with paired comparison designs, these models also allow for the estimation of the latent traits and person scores because the uniquenesses are generally non-zero in this design type. Finally, Thurstonian IRT models have been specified. These models can be used to estimate person scores in both paired comparison and ranking designs.

While the three types of models are technically straightforward, a major practical issue for applications of these models is that respondents have to perform many comparisons, even if the number of items is small. The number of necessary comparisons quickly becomes cumbersome and can escalate to an extent that may cause problems with data quality (respondent fatigue, cognitive load, motivation, noncompliance, etc.; see Sass et al., 2020). To illustrate, assume an investigation includes only *n* = 15 items. This already results in $\tilde{n}=105$ paired comparisons for every respondent. A typical psychological questionnaire can easily exceed 30 items, which would require a prohibitively large number of not-less-than 435 paired comparisons. Hence, there is a strong need in practical applications to reduce the number of items, as requiring that many decisions from participants is mentally exhausting and not feasible. Fortunately, a potential solution to this problem was proposed by Brown and Maydeu-Olivares (2011) with the multidimensional forced-choice format (MFC). The corresponding model will be called Thurstonian multidimensional block design (TMB) in the following sections.

### Thurstonian Multidimensional Block Design

To date, the MFC format and the TMB design described in this section are by far the most often applied ones in practice and research of Thurstonian modeling (e.g., Brown et al., 2017; Guenole et al., 2018; Ng et al., 2021; Salgado & Táuriz, 2014). The main idea of the TMB is as follows: Instead of using all possible paired comparisons, only a selection of item blocks is presented. Blocks can contain a number of *k* items, that is, a triplet has *k* = 3, a quad *k* = 4, and so on. The total number of items *n* must be divisible by *k* so that *p = n/k* blocks are presented to a respondent. This could be accomplished with any *k* > 1. Moreover, the number of latent traits must be at least *k* for the construction of multidimensional blocks. The TMB design is considered to be a generalization of the Thurstonian factor (and IRT) model because the properties of the latter models hold within each block, only moving from one to multiple blocks. However, it will be shown later that it is not a generalization but a simplification.

To date, the TMB design is used and discussed only in the IRT setting as the reparameterized Thurstonian factor model with unrestricted thresholds. However, it is not restricted to the IRT setting and can be used for all the aforementioned Thurstonian models. The model equations are also identical from a technical perspective, but there is a move from the single block to the multiple block perspective.

As an IRT example, consider *k* = 3 and *p* = 3 so that *n* = 9. In this case, the structured design matrix A would be

(31) $$A=\left(\begin{array}{ccccccccc} 1 & -1 & 0 & 0 & 0 & 0 & 0 & 0 & 0\\ 1 & 0 & -1 & 0 & 0 & 0 & 0 & 0 & 0\\ 0 & 1 & -1 & 0 & 0 & 0 & 0 & 0 & 0\\ 0 & 0 & 0 & 1 & -1 & 0 & 0 & 0 & 0\\ 0 & 0 & 0 & 1 & 0 & -1 & 0 & 0 & 0\\ 0 & 0 & 0 & 0 & 1 & -1 & 0 & 0 & 0\\ 0 & 0 & 0 & 0 & 0 & 0 & 1 & -1 & 0\\ 0 & 0 & 0 & 0 & 0 & 0 & 1 & 0 & -1\\ 0 & 0 & 0 & 0 & 0 & 0 & 0 & 1 & -1 \end{array}\right),$$

while the reparameterized matrix of loadings $\overset{⌣}{\Lambda }$ is

(32) $$\overset{⌣}{\Lambda }=A\Lambda =\left(\begin{array}{ccc} \lambda_{1} & -\lambda_{2} & 0\\ \lambda_{1} & 0 & -\lambda_{3}\\ 0 & \lambda_{2} & -\lambda_{3}\\ \lambda_{4} & -\lambda_{5} & 0\\ \lambda_{4} & 0 & -\lambda_{6}\\ 0 & \lambda_{5} & -\lambda_{6}\\ \lambda_{7} & -\lambda_{8} & 0\\ \lambda_{7} & 0 & -\lambda_{9}\\ 0 & \lambda_{8} & -\lambda_{9} \end{array}\right),$$

and the matrix of structured uniquenesses ${\overset{⌣}{\Psi }}^{2}=A\Psi^{2}$ is given by

(33) $$\begin{array}{c} {\overset{⌣}{\Psi }}^{2}=\\ \left(\begin{array}{ccccccccc} \psi_{1}^{2}+\psi_{2}^{2}+\omega_{1}^{2} & & & & & & & & \\ \psi_{1}^{2} & \psi_{1}^{2}+\psi_{3}^{2}+\omega_{2}^{2} & & & & & & & \\ -\psi_{2}^{2} & \psi_{3}^{2} & \psi_{2}^{2}+\psi_{3}^{2}+\omega_{3}^{2} & & & & & & \\ 0 & 0 & 0 & \psi_{4}^{2}+\psi_{5}^{2}+\omega_{4}^{2} & & & & & \\ 0 & 0 & 0 & \psi_{4}^{2} & \psi_{4}^{2}+\psi_{6}^{2}+\omega_{5}^{2} & & & & \\ 0 & 0 & 0 & -\psi_{5}^{2} & \psi_{6}^{2} & \psi_{5}^{2}+\psi_{6}^{2}+\omega_{6}^{2} & & & \\ 0 & 0 & 0 & 0 & 0 & 0 & \psi_{7}^{2}+\psi_{8}^{2}+\omega_{7}^{2} & & \\ 0 & 0 & 0 & 0 & 0 & 0 & \psi_{7}^{2} & \psi_{7}^{2}+\psi_{9}^{2}+\omega_{8}^{2} & \\ 0 & 0 & 0 & 0 & 0 & 0 & -\psi_{8}^{2} & \psi_{9}^{2} & \psi_{8}^{2}+\psi_{9}^{2}+\omega_{9}^{2} \end{array}\right). \end{array}$$

To identify the TMB design, the same identification constraints have to be used as was the case for the other Thurstonian models. However, they would need to be applied to every block. Also, for ranking data and blocks of size *k*, there are *r* (substitute *n* by *k* in Equation 13) redundancies among thresholds and tetrachoric correlations for each block. The number of redundancies *r* must be subtracted from the number of degrees of freedom reported by the structural equation modeling software, and fit indices must also be adjusted accordingly.

## Issues With Thurstonian Forced-Choice Models

There have been occasional critical reports in the literature in recent years concerning issues with the use of the MFC format and the TMB design, but most studies seem to add to the positive overall impression of the design’s potential. The more critical reports focus mainly on the issue of fakeability but also include a critical assessment of the use of mixed keyed blocks (e.g., Bürkner, 2022; Bürkner et al., 2019; Schulte et al., 2021). In this section, we will elaborate upon these issues that have previously been pointed out and add a number of additional problems that call the utility of current TMB designs and their application into question. This is done to identify and highlight current limitations and shortcomings on the one hand, while also pursuing the goal to constructively point out and sketch routes of improvement on the other. Overall, the issues with Thurstonian FC models as highlighted here will indicate its limited usefulness so far and lead to the conclusion that some of the results reported in the pertinent literature are probably biased.

A general and perhaps the most limiting factor for the application of the models in question is the number of comparisons that need to be performed if any of the non-block designs are used. Beyond the already mentioned practical limits imposed by the respondents’ abilities and motivation (see Sass et al., 2020), it is also important to note that even with limited information estimators, the use of many items can easily exceed the computational resources available (software capabilities, memory, and computational power). Such constraints negatively affect the applicability of any of the Thurstonian non-block designs considerably, even if cognitive load and motivation would not be an issue.

As a consequence of both the many paired comparisons a respondent would have to work on and the computational limitations, the use of the TMB design has been widespread. The number of blocks that can be presented in a TMB design is far less limiting than the number of paired comparisons. Because of its widespread use, the focus will henceforth be put on the TMB design and its issues. Hence, in what follows, a ranking model with e=0 and $\Omega^{2}=0$ is assumed.

The main question in using the TMB design is about which selection of items should be presented in which constellation of blocks, or in other words, what is the design of the assessment? The most often used block format is triplets. The number of all possible constellations of triplets for a set of *n* items is readily found, assuming *n* is a multiple of three. Let three items be drawn at a time without replacement and without considering the order. Let *k* be the vector of the number of items within each block. The multinomial coefficient provides the number of combinations as

(34) $$C_{n,k}=\left(\begin{array}{c} n\\ k_{1},k_{2},\cdots ,k_{p} \end{array}\right)=\frac{n!}{k_{1}!k_{2}!\cdots k_{p}!}.$$

In a typical design with only triplets, that is, all *k_i_* = 3, the number of combinations is

(35) $$C_{n,k}=\frac{n!}{3!3!\cdots 3!}=\frac{n!}{p\times 6}.$$

For *n* = 15, there are *p* = 5 triplets to be constructed, resulting in more than 43 billion possible ways to construct a test. This number gets considerably lower if we assume that *m* > 2 and that within each block, no trait is represented twice. Assuming *m* = 3 in the example, then for the first trait, there is one combination to sort the five items into five (yet empty) blocks. The order of the five items of the first trait is irrelevant. There are (*n*/3)! possible combinations to sort the *n*/3 items of Trait 2, and for each result, (*n*/3)! combinations to sort the *n*/3 items of Trait 3. Taken together, if no trait is considered twice within each block, then for *n* = 15 and *m* = 3, there are *p* = 5 triplets, for which $5!\times 5!=120^{2}=14,400$ combinations are possible. This illustrates that there are many possibilities to create a test in the framework of the TMB design. There are two main follow-up questions that will be addressed in the subsequent subsections: (a) Is there a best combination? and (b) does it even matter which combination is used?

### Issue 1: Precise Estimation vs. Fakeability

The first question, namely if there is a single best way to combine a given set of items and, if so, how to identify it, is not easy to answer. From previous studies, we know that there are better and worse ways to combine items into a block design, especially for the latent trait estimation (Brown & Maydeu-Olivares, 2011; Maydeu-Olivares & Brown, 2010). These are rooted in the difference values within or between factors. From Equations 23 and 24, it can be gathered that both thresholds and particularly the loadings of the items have a considerable impact on the ICCs and, therefore, also on the latent trait estimation. For example, if the difference between loadings of two items in a comparison is very small in a one-factor situation (i.e., *m* = 1), the influence of the factor is small or even zero in the case of equal loadings. To a certain degree, this is also true for comparisons when *m* = 2. However, as two different traits are considered, the effect on the two-dimensional surface is small only if the difference between loadings and the difference between the latent traits is simultaneously small. In addition, estimation is also dependent on the size of the correlation between traits (Brown & Maydeu-Olivares, 2011; Maydeu-Olivares & Brown, 2010). All these observations lead to the general recommendation to construct every block with at least one item with a positive loading and one item with a negative loading. This procedure, as can be expected from the explanation above, results in better trait estimation (less bias and therefore higher reliability) as was shown in several simulation studies (see Brown & Maydeu-Olivares, 2011).

However, this recommendation to construct every block with at least one item with a positive and one item with a negative loading provokes a serious issue: Blocks are more susceptible to faking by design. The recommendation to construct every block with at least one item with positive loading and one item with negative loading offers respondents an easy choice if they are inclined to respond in a socially favorable manner. In the dominance-response model, one item is strongly more or strongly less desirable than the others if the direction of the loading is different. The item that is chosen to be most or least appealing is the one that is most or least desirable. To illustrate, assume a triplet with the following statements for neuroticism, extraversion, and openness from the Big Five Triplets (BFT; Wetzel & Frick, 2020):

1. I have difficulty imagining things. (O−)
2. I like to talk to strangers. (E+)
3. I worry about things. (N+)

The loadings of the first statement will be negative, and the loadings of the last two statements will be positive. In a classical faking instruction for a job interview, the presentation of such a block likely results in the second statement to be chosen as “most like me.” Two of the three paired comparisons that can be derived from the block are prone to faking, and only one comparison yields useful information about the traits in question. This is not an arbitrarily exaggerated example for mixed keyed paired comparisons, and similar examples can easily be found (e.g., Anguiano-Carrasco et al., 2015; Bunji & Okada, 2020; Guenole et al., 2018; Walton et al., 2022)

As a consequence, constructing MFC tests that have differently keyed items within each block is not advisable and may be counterproductive with respect to the validity of the assessment (see also Bürkner et al., 2019; Schulte et al., 2021). The construction principle conflicts with the initial goal to reduce the effect of response biases and particularly faking when these response distortions are relevant issues in the given situation. That is, it would entirely defeat the purpose of using an FC assessment instead of ordinary Likert-type scales.

### Issue 2: Estimation of Specific (Block) Designs

The second follow-up question addressed here is, Does it matter which block combination is used? For the Thurstonian IRT models, where the latent traits are of interest, it is generally assumed that it does not matter which combination is used, since specific items are indicators of their respective traits. To show and illustrate that it indeed does matter which blocks are used, the associated assumptions that provide a foundation for this conclusion have to be specified first. Given are

1. A set of *n* items $t_{1},\ldots ,t_{n}$
2. A set of *m* latent traits $\eta_{1},\ldots ,\eta_{n}$
3. A mapping from $t_{1},\ldots ,t_{n}$ to $\eta_{1},\ldots ,\eta_{n}$ , that is, a matrix of loadings.

As a consequence of Assumptions 2 and 3, the simple Thurstonian model is disregarded here, and only the Thurstonian factor and IRT models are considered. However, the issue can also equivalently be expressed for simple Thurstonian models.

When a specific MFC test is constructed, and the focus is on the estimation based on the specific block design, then a choice among a large set of (several thousand if not billions) possible block designs that could be used for an assessment has to be made. The recommendation to only construct multidimensional block designs with at least one item with positive loading and one item with negative loading indeed effectively reduces the set of designs to choose from but was already dismissed as an advisable option in the previous subsection.

But what are the consequences of (randomly) choosing just any of the many admissible block designs? The most critical consequence of estimating a specific block design is that the specific MFC test (the operationalization of the assumed theoretical model) determines the test model that the data are applied to in a confirmatory sense (the specific block design), and not the other way around. To illustrate this intricate issue with a simplified example, the performance of a simulation study and data generation of one person is considered. For illustrative purposes, the following setting may be given: *n* = 9, *m* = 3, and *p* = 3. Data for one respondent will be generated from the Thurstonian factor model (Equation 10), with $\mu_{t}=\left(-.1,.3,.2,-.2,.3,-.1,.2,-.1,0\right)$ , as well as $\eta =\left(\eta_{1},\eta_{2},\eta_{3}\right)=\left(-.5,.1,.6\right)$ and

(36) $$\Lambda =\left(\begin{array}{ccc} -.6 & 0 & 0\\ .7 & 0 & 0\\ .5 & 0 & 0\\ 0 & -.6 & 0\\ 0 & .6 & 0\\ 0 & .8 & 0\\ 0 & 0 & .8\\ 0 & 0 & -.6\\ 0 & 0 & .4 \end{array}\right).$$

The error terms ε and **e** are ignored here for simplicity, which results in deterministic simulations. It is shown that for these conditions, the data as well as the results depend heavily on the blocks chosen. First, consider a full design (all 36 paired comparisons). For demonstration purposes, we will focus on the ipsative ranking of the traits within a respondent and add the estimated MAP scores by Mplus. The ipsative order for the full design for the simulated data is $\left(\eta_{3},\eta_{2},\eta_{1}\right)$ , that is, the third trait would be rated as most fitting, the second trait as the second best, and the first trait as the least-fitting descriptor of oneself. This corresponds to the true vector of trait scores η=(−.5,.1,.6) . Now, consider a multidimensional block design with exactly one item having a negative loading in each block as recommended in the literature (e.g., Brown & Maydeu-Olivares, 2011). The design matrix A for such a design could be

(37) $$A_{1}=\left(\begin{array}{ccccccccc} 1 & 0 & 0 & 0 & -1 & 0 & 0 & 0 & 0\\ 1 & 0 & 0 & 0 & 0 & 0 & 0 & 0 & -1\\ 0 & 0 & 0 & 0 & 1 & 0 & 0 & 0 & -1\\ 0 & 1 & 0 & -1 & 0 & 0 & 0 & 0 & 0\\ 0 & 1 & 0 & 0 & 0 & 0 & -1 & 0 & 0\\ 0 & 0 & 0 & 1 & 0 & 0 & -1 & 0 & 0\\ 0 & 0 & 1 & 0 & 0 & -1 & 0 & 0 & 0\\ 0 & 0 & 1 & 0 & 0 & 0 & 0 & -1 & 0\\ 0 & 0 & 0 & 0 & 0 & 1 & 0 & -1 & 0 \end{array}\right).$$

This corresponds to blocks B_1_ = (1,5,9), B_2_ = (2,4,7), and B_3_ = (3,6,8), for which

(38) $$y^{*}=A(\mu_{t}+\Lambda \eta )=\left(\begin{array}{c} -.16\\ -.04\\ .12\\ .21\\ -.73\\ -.94\\ -.03\\ .41\\ .44 \end{array}\right)\Rightarrow y=\left(\begin{array}{c} y_{15}\\ y_{19}\\ y_{59}\\ y_{24}\\ y_{27}\\ y_{47}\\ y_{36}\\ y_{38}\\ y_{68} \end{array}\right)=\left(\begin{array}{c} 0\\ 0\\ 1\\ 1\\ 0\\ 0\\ 0\\ 1\\ 1 \end{array}\right).$$

From Equation 38, it can be gathered that for the first block, Item 1 (Trait 1) is second in both paired comparisons, Item 5 (Trait 2) is chosen first in both paired comparisons, and Item 9 (Trait 3) is chosen over Item 1 but not Item 5. Define $\eta_{\mathrm{gh}}$ , where *g* is the number of the trait (1, 2, and 3), and *h* is the block considered (1, 2, and 3). As a result, the ranks for the first block are $\left(\eta_{11}=3,\eta_{21}=1,\eta_{31}=2\right)$ . For the second block, it is $\left(\eta_{12}=2,\eta_{22}=3,\eta_{32}=1\right)$ , and for the third, it is $\left(\eta_{13}=2,\eta_{23}=1,\eta_{33}=3\right)$ , respectively. If the traits are ranked over blocks, the mean ranks are 2.33 for the first trait, 1.67 for the second trait, and 2 for the third. Hence, the order of traits would be $\left(\eta_{2},\eta_{3},\eta_{1}\right)$ . The same order is true for the MAP scores $\eta_{\mathrm{MAP}}=\left(0.50,1.30,0.87\right)$ . This does not correspond to the true scores η=(−.5,.1,.6) according to which the order should be $\left(\eta_{3},\eta_{2},\eta_{1}\right)$ . Now let us assume a different block design is given in the same situation that may have alternatively be chosen:

(39) $$A_{2}=\left(\begin{array}{ccccccccc} 1 & 0 & 0 & 0 & 0 & -1 & 0 & 0 & 0\\ 1 & 0 & 0 & 0 & 0 & 0 & 0 & 0 & -1\\ 0 & 0 & 0 & 0 & 0 & 1 & 0 & 0 & -1\\ 0 & 1 & 0 & -1 & 0 & 0 & 0 & 0 & 0\\ 0 & 1 & 0 & 0 & 0 & 0 & -1 & 0 & 0\\ 0 & 0 & 0 & 1 & 0 & 0 & -1 & 0 & 0\\ 0 & 0 & 1 & 0 & -1 & 0 & 0 & 0 & 0\\ 0 & 0 & 1 & 0 & 0 & 0 & 0 & -1 & 0\\ 0 & 0 & 0 & 0 & 1 & 0 & 0 & -1 & 0 \end{array}\right).$$

This corresponds to blocks B_1_ = (1,6,9), B_2_ = (2,4,7), and B_3_ = (3,5,8). Items are only slightly shuffled as compared to the first block design. More specifically, only Items 5 and 6 are switched. It follows

(40) $$y^{*}=\left(\begin{array}{c} .22\\ -.04\\ -.26\\ .21\\ -.73\\ -.94\\ -.41\\ .41\\ .82 \end{array}\right)\Rightarrow y=\left(\begin{array}{c} y_{16}\\ y_{19}\\ y_{69}\\ y_{24}\\ y_{27}\\ y_{47}\\ y_{35}\\ y_{38}\\ y_{58} \end{array}\right)=\left(\begin{array}{c} 1\\ 0\\ 0\\ 1\\ 0\\ 0\\ 0\\ 1\\ 1 \end{array}\right).$$

Again, if the traits are ranked over blocks, the mean ranks are 2 for the first trait, 2.33 for the second, and 1.67 for the third. The order would therefore be $\left(\eta_{3},\eta_{1},\eta_{2}\right)$ . Again, the same order is true for the MAP scores $\eta_{\mathrm{MAP}}=\left(0.69,0.26,1.33\right)$ . Let us assume a third different block design in the same situation that may have alternatively be chosen:

(41) $$A_{3}=\left(\begin{array}{ccccccccc} 1 & 0 & 0 & 0 & -1 & 0 & 0 & 0 & 0\\ 1 & 0 & 0 & 0 & 0 & 0 & -1 & 0 & 0\\ 0 & 0 & 0 & 0 & 1 & 0 & -1 & 0 & 0\\ 0 & 1 & 0 & 0 & 0 & -1 & 0 & 0 & 0\\ 0 & 1 & 0 & 0 & 0 & 0 & 0 & -1 & 0\\ 0 & 0 & 0 & 0 & 0 & 1 & 0 & -1 & 0\\ 0 & 0 & 1 & -1 & 0 & 0 & 0 & 0 & 0\\ 0 & 0 & 1 & 0 & 0 & 0 & 0 & 0 & -1\\ 0 & 0 & 0 & 1 & 0 & 0 & 0 & 0 & -1 \end{array}\right).$$

The design includes blocks B_1_ = (1,5,7), B_2_ = (2,6,8), and B_3_ = (3,4,9). As can be seen, even more items have been shuffled across blocks, but it is just another possible block design in the given situation. Now it is

(42) $$y^{*}=\left(\begin{array}{c} -.16\\ -.48\\ -.32\\ -.03\\ .41\\ .44\\ .21\\ -.29\\ -.50 \end{array}\right)\Rightarrow y=\left(\begin{array}{c} y_{15}\\ y_{17}\\ y_{57}\\ y_{26}\\ y_{28}\\ y_{68}\\ y_{34}\\ y_{39}\\ y_{49} \end{array}\right)=\left(\begin{array}{c} 0\\ 0\\ 0\\ 0\\ 1\\ 1\\ 1\\ 0\\ 0 \end{array}\right).$$

If the traits are ranked within each block again, the mean ranks are 2.33 for the first, 2 for the second, and 1.67 for the third trait, and the order would be $\left(\eta_{3},\eta_{2},\eta_{1}\right)$ . The MAP scores yield yet again the same order $\eta_{\mathrm{MAP}}=\left(0.42,0.88,1.19\right)$ .

What this elaborate example shows is that the simulated pattern of paired comparisons actually yields different results for the simulated preferences of traits for all three block designs. Note that this is true even without considering error terms. The only difference between the three designs is the block design as represented by matrix A , and all other fundamental parameters are identical. That is, ceteris paribus, the second trait could be modeled as the most preferred, the least preferred, or in between just by choosing a specific design. Of course, this illustration is limited to a specific situation with *n* = 9, *m* = 3, and *p* = 3. Multidimensional blocks without the loading recommendation or unidimensional paired comparisons are not considered.

Nevertheless, the example shows that the choice of a specific block design can indeed matter, and it can result in remarkable differences (not only) in simulated data. This implies that simulated data can already be biased. Given these circumstances, the result almost certainly is that the estimation of the model and the calculation of factor scores can also be biased. To illustrate this with an empirical example, data from the study of Jansen and Schulze (2023a) are reanalyzed.

In this study, 15 positively keyed adjectives that are all intended to assess conscientiousness were used, and the social desirability scores of these items were estimated. Participants were presented with the entire set of 105 possible paired comparisons and a block of all 15 items. This amounts to the fact that data for the full design is available. In the present reanalysis, data is considered for ranking. If three different designs were chosen, the results differed not only between the three designs but also in comparison to the parameter estimation by the full design. Consider the arbitrarily chosen designs:

Blocks are given by

1. Items [1,2,3], [4,5,6], [7,8,9], [10,11,12], and [13,14,15]
2. Items [1,5,6], [2,4,11], [7,12,15], [3,8,10], and [9,13,14]
3. Items [1,9,14], [4,8,12], [2,5,7], [6,10,15], and [3,11,13]

The relative bias of the factor loadings on social desirability and item utilities of a Thurstonian factor model are given in Table 1. As can be seen, the estimated factor loadings and item utilities from the block designs deviate strongly from the estimates based on the full design. These relative biases appear to be beyond acceptable. In addition, the correlation between factor scores (estimated with the corresponding Thurstonian IRT models) of the block designs and the full design are *r_Fd1_* = −.42, *r_Fd2_* = .91, and *r_Fd3_* = .87, respectively. Interestingly, the block design with the lowest mean relative bias for loading and utility estimates is the design with the lowest correlation for estimated factor scores.

**Table 1.** The Relative Bias of the Three Block Designs Compared to the Full Design.

Item/parameter	Loadings			Utilities		
	D1	D2	D3	D1	D2	D3
I01*	0.00	0.00	0.00	0.00	0.00	0.00
I02	−0.08	−0.69	−0.24	0.25	−2.68	−1.33
I03	−0.64	−0.70	−0.37	0.45	−0.55	−1.13
I04*	−0.48	−0.48	−0.48	−1.00	−1.00	−1.00
I05	−0.53	6.60	−0.17	−2.61	10.55	−1.58
I06	−0.76	9.35	−0.54	−2.04	10.56	−0.83
I07*	−0.16	−0.16	−0.16	−1.00	−1.00	−1.00
I08	1.12	−1.14	−1.04	−1.64	−1.28	−3.71
I09	−0.68	−0.57	−0.08	−2.11	−3.16	0.04
I10*	−0.58	−0.58	−0.58	−1.00	−1.00	−1.00
I11	−0.61	−0.45	−0.45	−0.49	−0.75	−0.71
I12	−0.61	−0.20	−0.37	−0.66	−0.76	−1.52
I13*	−0.52	−0.52	−0.52	−1.00	−1.00	−1.00
I14	0.03	−0.74	0.02	18.73	20.68	−0.52
I15	0.24	−0.38	−1.07	−1.63	−1.01	−0.80
*M*	−0.28	0.62	−0.40	0.28	1.84	−1.07

*Note.* D1, D2, and D3 denote designs 1, 2, and 3, respectively. Values with an asterisk “*” are fixed values.

To mitigate or even remove the influence of the choice of a specific block design, the possibility of the many different designs that can be focused on needs to be eliminated. The solution to this appears to be almost trivial: The one (and only) design that should be in focus, especially for data generation and simulation studies, must be the design where all paired comparisons are considered (i.e., the full design). Given any item set and any set of traits, the full design is always unique.

An important corollary is that data should always be generated with the full design in any simulation study with Thurstonian models. Previous simulation studies did generate data from the specific block designs, however. The block designs were tested (and compared) on the basis of the resulting data (e.g., Brown & Maydeu-Olivares, 2011; Bürkner et al., 2019; Schulte et al., 2021). The same is true for any simulation study with the R package thurstonianIRT (Bürkner, 2019) because it also implements the simulation by each specific design. As we have illustrated, chances are high that these simulation studies provide biased results. An update on the results reported in previous simulation studies and further evidence that the simulations were indeed biased is given by Jansen and Schulze (2023b).

### Issue 3: Identification Constraints in Block Designs

The reanalysis in the previous subsection also reveals another issue with the TMB: Identification constraints need to be applied to every block, thereby setting different scales for the parameters. While the chosen identification constraints are statistically irrelevant (see Maydeu-Olivares & Böckenholt, 2005; Maydeu-Olivares & Brown, 2010), they are highly relevant for the specific estimates as they set the scale. This can be illustrated with a non-IRT TMB design. Assume that the focus is on estimating the means of the latent utilities of the items for item scaling. Also assume *n* = 9, *m* = 3, *p* = 3, (again) $\mu_{t}=\left(-.1,.3,.2,-.1,.2,.1,.2,-.1,0\right)$ , and Λ from Equation 36. Then with blocks B_1_ = (1,5,7), B_2_ = (2,6,8), and B_3_ = (3,4,9), the question is, which of the mean latent utilities will be fixed for identification to what value? A common approach would be to fix, for example, the last item per block to $\mu_{t}=0$ (Maydeu-Olivares & Böckenholt, 2005). Suppose parameter estimation would be perfect, then the result would be $\mu_{t_{\mathrm{est}}}=\left(-.3,.4,.2,-.1,0,.2,0^{*},0^{*},0^{*}\right)$ , where the values with an asterisk are fixed values. In the original data, we had $\mu_{3}=.2$ , $\mu_{5}=.2$ , and $\mu_{6}=.1$ , which corresponds to Items 3 and 5 having the same and Item 6 having a smaller mean utility. In contrast, the example estimates are $\mu_{3}=.2$ , $\mu_{5}=0$ , and $\mu_{6}=.2$ , which corresponds to Items 3 and 6 having the same and Item 5 having a smaller mean utility.

The core issue is that no reference point for any of the blocks exists. Without any further information, it is impossible to estimate all latent utility means simultaneously. Arbitrarily chosen identification constraints severely limit the use of the estimates. When identification constraints are arbitrarily chosen, they enforce an equivalence between two or more parameters that would only be valid, if further information was available to justify the equivalence. This is probably a rare rather than a common case in applications. Again, it is referred to Jansen and Schulze (2023a), where the goal was to estimate social desirability values for each item. With the estimated values from a full design, the matching of similar or equally desirable items per block was possible. This is the case because only one item utility needed to be fixed, and the other utilities are estimated in relation to the fixed parameter. Assume a block design would be used. The arbitrary identification constraints within each block would identify the design. However, knowledge about the relationship between the latent utility means that are fixed for identification (differences and dispersion) is needed to justify the constraints. Fixing one latent utility mean per block to zero amounts to the theoretical assumption that these fixed latent utility means are indeed equal. If the assumption is untrue, then using the constraints results in invalid estimates for between-block relations. As a consequence, the estimated parameters have very limited usefulness. Furthermore, the procedure can result in biased estimation for most model parameters. It can be gleaned from the analyses as presented in Table 1, for example, that the loadings and utilities are biased except for the parameters of the first item, which was used for identification constraints also in the full design.

### Issue 4: Estimation of Reliability and Recovery of Latent Traits

In empirical studies, the true latent trait scores are practically never known. The recovery and reliability of these scores can only be estimated using the respondents’ pattern of binary outcome responses. As binary indicators are used and an IRT setting is given, with limited information estimators, factor scores are best estimated using the MAP estimation.

The actual recovery is defined as the correlation between the true trait scores and their estimates. The empirical recovery can be calculated as stated in Equations 28 and 30 by using the square of the reliability. Previous simulation studies (Brown & Maydeu-Olivares, 2011; Maydeu-Olivares & Brown, 2010) indicate that the recovery of the latent traits is only reliable if positively and negatively keyed items are used within blocks. For designs with items keyed in only one direction, the estimation of the empirical recovery is not recommended by Brown and Maydeu-Olivares (2011). To understand why, it is necessary to realize that the IRT factor scores are dependent on the test information function $I^{j}\left(\eta \right)$ for trait *j*. The test information function is computed by using the item information functions $I_{l}^{j}\left(\eta \right)$ , where the *l* items are the binary outcomes. The information provided by one binary outcome for two traits *a* and *b* is (see Ackerman, 2005; Brown & Maydeu-Olivares, 2011)

(43) $$I_{l}^{a}\left(\eta_{a},\eta_{b}\right)=\frac{{\left[\beta_{i}-\beta_{k}\mathrm{corr}\left(\eta_{a},\eta_{b}\right)\right]}^{2}{\left[\phi \left(\alpha_{l}+\beta_{i}\eta_{a}-\beta_{k}\eta_{b}\right)\right]}^{2}}{P_{l}\left(\eta_{a},\eta_{b}\right)\left[1-P_{l}\left(\eta_{a},\eta_{b}\right)\right]}$$

and

(44) $$I_{l}^{b}\left(\eta_{a},\eta_{b}\right)=\frac{{\left[-\beta_{k}+\beta_{i}\mathrm{corr}\left(\eta_{a},\eta_{b}\right)\right]}^{2}{\left[\phi \left(\alpha_{l}+\beta_{i}\eta_{a}-\beta_{k}\eta_{b}\right)\right]}^{2}}{P_{l}\left(\eta_{a},\eta_{b}\right)\left[1-P_{l}\left(\eta_{a},\eta_{b}\right)\right]},$$

where $\alpha_{i}$ and $\beta_{i}$ are the intercepts and slopes of the binary variables and the items, respectively, and $P_{l}\left(\eta_{a},\eta_{b}\right)=P(y_{l}=1|\eta_{a},\eta_{b})$ . The item information depends on the difference between slopes $\beta_{i}$ , which is small if the difference between loadings is small (see Equation 25). The standard errors of the MAP estimates are estimated by the reciprocals of the square root of the posterior test information $I_{p}^{j}\left(\eta \right)$ :

(45) $$\mathrm{SE}({\hat{\eta }}_{j})=\frac{1}{\sqrt{I_{p}^{j}\left(\eta \right)}}.$$

An additional issue is that the empirical recovery overestimates the actual recovery, especially in cases where the difference between loadings is small (see also Yousfi, 2019). However, Brown and Maydeu-Olivares (2011) provided evidence from a simulation study that the empirical recovery underestimates the actual recovery or is close to it. We suspect that this discrepancy is also a result of the simulation from the block designs and not the full design (see the previous subsection).

To illustrate, a small simulation study for full designs was conducted. The results of the simulation study and all code to replicate the results are made available on OSF (Supplemental Material: https://osf.io/aqrhb/). We simulated 100 data sets with 1,000 respondents from full Thurstonian factor models (Equation 10) with *m* = 3 uncorrelated factors and six items per factor (*n* = 18). The first design included only items with positive item loadings that were drawn from a uniform distribution at the interval [0.3, 0.9]. The intercepts were drawn from the interval [−1, 1]. The second design had the same specifications; only half of the loadings were negative. The results on the MAP scores from Mplus (L. K. Muthén & Muthén, 1998–2022) and corrections via genuine likelihood (Yousfi, 2019) as well as the correlation with the true scores are given in Table 2.

**Table 2.** Results for the Latent Trait Recoveries of the Simulation Study.

Keyed direction of items	Actual			Empirical		
	Trait 1	Trait 2	Trait 3	Trait 1	Trait 2	Trait 3
Local independence						
+	.730	.743	.772	.934	.936	.944
+/−	.887	.894	.916	.974	.975	.978
Yousfi (2019)						
+	.750	.762	.802	.623	.654	.679
+/−	.878	.884	.911	.733	.772	.794

*Note.* The actual recovery is computed as the correlation between true scores and MAP score estimates. The empirical recovery is calculated using the square root of Equation 29. + = positive loadings only, +/− positive and negative loadings.

As can be seen in Table 2, the actual recoveries are relatively low for designs where items load only in one direction and reliabilities (square of the recoveries) are not within an acceptable range. In designs including items loading in both directions, the information provided by each paired comparison is larger, and latent trait recovery is much better. In addition, we see that the empirical recovery by Brown and Maydeu-Olivares (2018) overestimates the actual recovery, especially if only positively keyed items are used. The genuine likelihood approach does not change the results on the real recovery substantially but underestimates the real recovery.

In sum, it was illustrated that latent trait recovery is problematic and generally seems to be overestimated (local independence) or underestimated (genuine likelihood) in Thurstonian FC modeling. In addition, in model designs where only blocks are used, this issue is aggravated. Past simulation studies already indicated lower reliability in Thurstonian modeling than rating scale reliabilities (e.g., Wetzel & Frick, 2020). Finally, in IRT settings, the reliability of trait scores depends also on the respondents’ location on the traits.

## Discussion

The present article provided an overview of the status and a number of issues with current linear factor analytic Thurstonian FC models and their variants. The main reasons why these models have been introduced is because they are connected to concerns about the validity of responses in the assessment of constructs from the realm of typical psychological behavior. Threats to the validity of responses can arise from unintentional response distortions and biases as well as intentional response tampering such as faking. The use of this class of models is associated with the expectation that they provide solutions to these long-standing issues in psychological assessment. Recent developments of Thurstonian FC models also go beyond an adaptation of Thurstone’s original models used in psychophysics and offer modern model testing and parameter estimation. Generally, these models are suitable from a dominance-response perspective for flexible modeling of binary responses to items from FC questionnaires. Thurstonian FC modeling can focus on the estimation of latent utilities of items or the estimation of latent factor scores of respondents, thereby providing a framework that includes both item and person scaling. The models encompass both CFA and IRT procedures for these purposes. With software templates and packages available, the estimation can be straightforwardly implemented and, with limited information estimation, is fast, especially compared to full information maximum likelihood estimation. All these highly attractive features of Thurstonian FC models indeed justify the rise in publications and applications with this method in the last 15 years.

As was elaborated in this article, though, it is not entirely clear yet whether the use of Thurstonian FC models, as they are currently applied, actually leads to enhanced validity of the assessments. The potential benefits are also accompanied by a number of theoretical and practical issues that threaten or at least hamper the realization of the benefits. One of the more obvious difficulties for applications of the Thurstonian FC models is that the use of the full designs, which is where all possible paired comparisons are needed, is infeasible. This is true for both the estimation of the models as well as the respondents that need to perform all paired comparisons. Hence, the design of the assessment is one of the major topics of research on Thurstonian FC models. Some proposals to make the application of Thurstonian FC models feasible with triplets, for example, have already been made and implemented. In the present article, four problematic areas that are given with the proposed solutions were addressed and scrutinized. Some of these issues have implications mainly for the practical use of the Thurstonian FC models; others also pertain to the design of simulation studies and the interpretation of their results. For example, it was shown that the MFC format with its constraint to multidimensional blocks and the estimation with the TMB designs can lead to biased results. Also, the recommendation to use both positively and negatively keyed items within each block results in potentially highly fakable blocks. Therefore, it undermines the main advantage to potentially reduce faking and response biases (see also Bürkner, 2022; Bürkner et al., 2019), which has been the motivation to use FC assessments in the first place.

Given these results, a change in recommendations for the assessment design with Thurstonian FC model is derived, namely not to exclusively use differently keyed items in blocks when the MFC format or any other block format is used. It should be noted, though, that following this recommendation is expected to lead to responses less prone to faking but at the cost of less-reliable estimations (e.g., Brown & Maydeu-Olivares, 2011; Bürkner, 2022; Maydeu-Olivares & Brown, 2010; Schulte et al., 2021).

In addition, this also draws attention to the fact that fakeability and/or desirability of items and item blocks should explicitly be accounted for. It can be assumed that blocks with items that have different scores on a desirability scale are easily fakable, even when the items in a block are keyed in the same direction. If faking is of concern when using the Thurstonian FC models, it is therefore recommended as a first step to carefully estimate the desirability scores of items or item blocks before assembling items into blocks. This can be done with the simple Thurstonian or Thurstonian factor model (e.g., Jansen & Schulze, 2023a) or using a rating scale approach (e.g., Wetzel & Frick, 2020). A second step could be to use the recently introduced faking mixture model to estimate the fakeability of item blocks (Frick, 2022) after these blocks were matched for desirability.

With respect to simulation studies with Thurstonian FC models, the issues identified here lead the recommendation that simulated data should always be derived from the full design. If any Thurstonian block design is used, paired comparisons that are simulated but not of interest can just be discarded. However, it was shown that the use of any TMB is problematic and leads to biased results. This is also and particularly true since, for each block, arbitrary identification constraints must be applied. To overcome this issue, it is recommended to apply the identification constraints in relation to one another or to establish the relation or linkage between the blocks by other justifiable means. This may result in longer yet still feasible assessments, however (such a linkage is proposed by Jansen & Schulze, 2023b).

Overall, the Thurstonian FC models are a very important recent psychometric development for the assessment of a typical psychological behavior. The fact that some issues and problem areas as explicated in this article still prevail for the models in their current status does not imply at all that the use of the models would be not advisable. Research on the Thurstonian FC models is still a vibrant field, and it appears to be quite reasonable to expect solutions, or at least significant improvements, in the problem areas as outlined in this article. Until then, careful interpretation of results from the use of Thurstonian FC models taking the issues into account appears to be advisable.
